# Predictors of Dysplasia in Oral Submucous Fibrosis: A Retrospective Observational Study

**DOI:** 10.7759/cureus.78481

**Published:** 2025-02-04

**Authors:** Mrinal Shete, Priyanka P Kamble, Kshitija Patil, Basavaraj T Bhagawati, Ujwala Brahmankar, Manish Sharma

**Affiliations:** 1 Department of Oral and Maxillofacial Pathology, D Y Patil Dental School, Pune, IND; 2 Department of Oral and Maxillofacial Pathology, Jawahar Medical Foundation's Annasaheb Chudaman Patil Memorial Dental College, Dhule, IND; 3 Department of Oral and Maxillofacial Surgery, Jawahar Medical Foundation's Annasaheb Chudaman Patil Memorial Dental College, Dhule, IND; 4 Department of Oral Medicine and Radiology, Dashmesh Institute of Research and Dental Sciences, Faridkot, IND; 5 Department of Oral Medicine and Radiology, Jawahar Medical Foundation's Annasaheb Chudaman Patil Memorial Dental College, Dhule, IND

**Keywords:** dysplasia, malignant, oral submucous fibrosis, predictors, transformation

## Abstract

Introduction: Oral submucous fibrosis (OSMF) is a progressive, potentially malignant disorder often associated with areca nut and tobacco consumption. Dysplasia, a key predictor of malignant transformation, is poorly understood in terms of its association with clinical and histopathological factors. This study aimed to identify the clinical and histopathological predictors of dysplasia in OSMF to enhance diagnostic accuracy and risk stratification.

Materials and methods: This retrospective observational study was conducted in the Department of Oral Pathology. A total of 545 case records were screened, and 120 histopathologically confirmed OSMF cases that met the strict inclusion and exclusion criteria were analyzed. Clinical parameters such as mouth opening, clinical staging, lesion site, and history of tobacco, smoking, and alcohol use were documented. Histopathological variables including epithelial keratinization, dysplasia, and epithelial thickness were evaluated. Statistical analyses, including logistic regression and discriminant analysis, were performed to identify significant predictors.

Results: Dysplasia was observed in 48 (40%) participants. Alcohol consumption was the strongest predictor of dysplasia (p = 0.0001), followed by epithelial thinning (p = 0.001), and smoking (p = 0.012). Reduced mouth opening was also significantly associated with dysplasia (p = 0.001). Keratinization and burning sensation were common but demonstrated minimal contribution to dysplasia risk. Discriminant analysis revealed that alcohol frequency and epithelial thinning were the most influential factors in distinguishing OSMF cases with dysplasia from those without dysplasia.

Conclusion: This study identifies alcohol consumption, epithelial thinning, and smoking as significant predictors of dysplasia in patients with OSMF. Routine histopathological evaluations and targeted interventions addressing modifiable risk factors, particularly alcohol consumption and smoking, are critical for mitigating the risks of malignant transformation.

## Introduction

Oral submucous fibrosis (OSMF) is a progressive and potentially malignant condition marked by significant morphological (blanching of mucosa and epithelial atrophy with or without dysplasia) and physiological modifications (burning sensation, reduced mouth opening, and mucosal dryness) in the oral mucosa, predominantly affecting populations in South and Southeast Asia [1,2]. Epidemiological studies have consistently indicated a striking prevalence rate ranging from 0.2% to 7.6% among Indian populations, with elevated incidence rates noted in geographic areas characterized by high consumption of areca nuts and tobacco [3]. The multifaceted etiopathogenesis of OSMF involves intricate interactions between environmental, genetic, and nutritional factors, with areca nut chewing identified as the principal etiological trigger [4].

Extensive longitudinal research has definitively demonstrated significant risk stratification for malignant transformation in cases of OSMF, with anticipated probabilities of oral cancer development ranging from 7% to 30% over prolonged observational periods [5,6]. The advancing cellular and molecular mechanisms encompass persistent inflammatory responses, oxidative stress, and progressive collagen hyalinization, which progressively undermine the structural integrity of tissues and regulatory cellular mechanisms [7,8]. Clinical indicators such as increasing mucosal stiffness, limited mouth opening, fibrous bands, and pigmentation serve as essential diagnostic markers for the likelihood of malignant progression [9].

Histopathological evaluation is essential for the early detection of dysplasia, necessitating thorough examination of alterations in cellular architecture, nuclear pleomorphism, and abnormalities in epithelial stratification [10]. OSMF cases exhibit a high prevalence of epithelial dysplasia, with dysplasia detected in approximately 10%-15% of early-stage OSMF cases and up to 30%-50% in advanced stages [9]. The prompt recognition of dysplastic alterations is crucial, as intervention at the nascent stages may significantly reduce the risk of malignant transformation. Molecular biomarkers such as p53 mutations, irregularities in cell cycle regulation, and the expression of inflammatory cytokines enhance the diagnostic accuracy for a more thorough risk evaluation [11,12]. This retrospective observational study aimed to explore the clinical and histopathological indicators that contribute to a deeper understanding of the mechanisms underlying dysplastic progression in OSMF, with the potential to establish more nuanced diagnostic and prognostic stratification frameworks for populations at elevated risk.

## Materials and methods

Study design and setting

This retrospective observational study utilized archival records from January 2015 to June 2024, obtained from the Department of Oral Pathology at Jawahar Medical Foundation's Annasaheb Chudaman Patil Memorial Dental College, Dhule. The investigation was conducted from October 2024 to December 2024, following approval from the Institutional Ethics Committee (EC/NEW/INST/2022/2959/2024/30). The study rigorously adhered to the ethical principles outlined in the Declaration of Helsinki, ensuring ethical integrity and standardized research protocols throughout the investigation.

Sample size estimation

The G*Power software (Heinrich Heine Universität Düsseldorf, Düsseldorf, Germany) was used to determine the appropriate sample size. Considering an 85% study power and a 5% alpha error, the required sample size was calculated to be 120 cases of OSMF, based on the estimated proportion of dysplasia in OSMF ranging between 15% and 20% [5].

Inclusion and exclusion criteria

The inclusion criteria for this study were as follows: clinical and histopathologically confirmed cases of OSMF, with complete and detailed case records available for demographic, clinical, and histopathological evaluations. Patients of all age groups and of both sexes were included to ensure a representative sample. Patients with sufficient clinical documentation, including information on mouth opening (in millimeters), clinical staging of OSMF, lesion site, burning sensation of the mucosa, and habits such as tobacco, alcohol, and smoking, were considered eligible. Additionally, histopathological records containing details of a sufficient thickness of the epithelium were mandatory for inclusion. Exclusion criteria included incomplete or missing records, particularly those lacking clinical or histopathological data essential for the analysis. Patients with a history of prior surgical or medical treatment for OSMF or other oral lesions were excluded to avoid confounding results. Patients diagnosed with systemic diseases, malignancies, or other potentially malignant oral disorders such as leukoplakia or erythroplakia coexisting with OSMF were also excluded. These criteria ensured a focused and homogeneous sample for evaluating the clinical and histopathological predictors of dysplasia in OSMF.

Methodology

**Figure 1:**
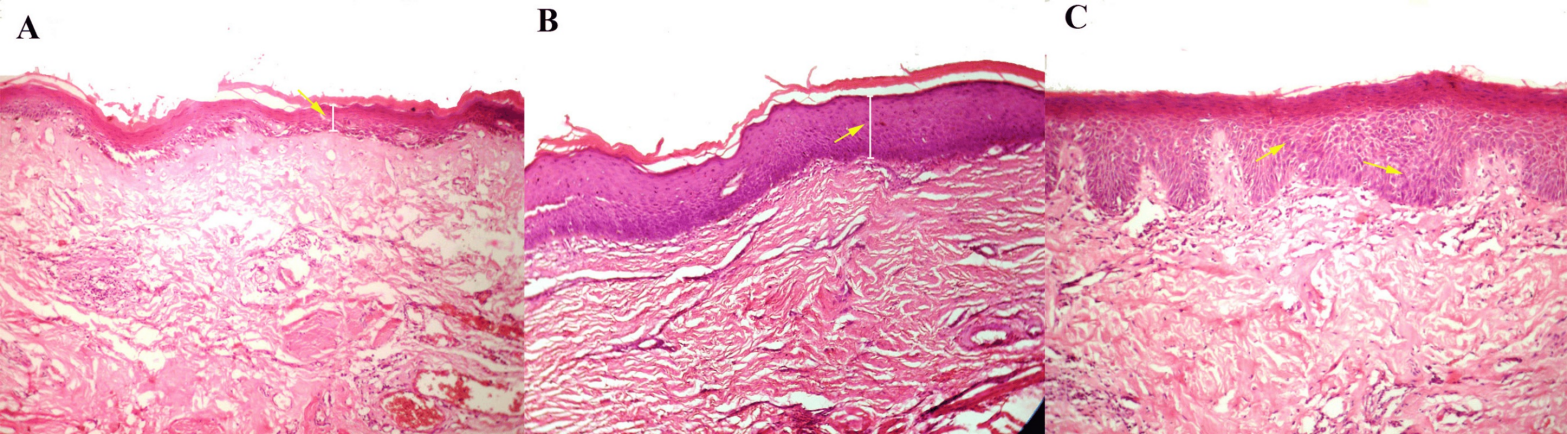
Histopathology of OSMF. (A) Thin epithelium. (B) Thick epithelium with keratinization. (C) Epithelium with dysplasia. Hematoxylin and eosin-stained sections at a magnification of 20X. Image credit: This figure is an original creation of the authors.

A total of 545 case records of OSMF were screened from the archives of the department, and inclusion and exclusion criteria were applied to select eligible cases. This yielded a final sample size of 120 cases for analysis. Each case was thoroughly evaluated for a variety of study variables, including demographic data (age and sex), clinical parameters (mouth opening in millimeters) [13], burning sensation of mucosa scored on a Visual Analog Scale (VAS), clinical stage of OSMF, site of the lesion, history of tobacco, alcohol, and smoking, and histopathological features (histopathological grade of OSMF [10], epithelial keratinization, epithelial dysplasia, and epithelial thickness) (Figure 1).

Statistical analysis

Statistical analyses were performed using the IBM SPSS Statistics for Windows, Version 23.0 (Released 2015; IBM Corp., Armonk, NY, United States). The Shapiro-Wilk test was used to confirm the normality of the data distribution. Categorical data are presented as frequencies and percentages, while continuous data are summarized as mean ± standard deviation. Logistic regression analysis was performed to identify clinical and histopathological predictors of dysplasia. Additionally, discriminant analysis was applied to explore the relationships and associations between categorical variables, providing insights into potential patterns.

## Results

The descriptive characteristics of the study population are summarized in Table 1. Among the 120 patients analyzed, 90 (75%) were males, and 30 (25%) were females. A total of 102 (85%) participants reported a history of tobacco use, and 66 (55%) were smokers. Alcohol consumption was seen in half of the participants. Burning sensation, a common symptom in OSMF, was reported in 72 (60%) participants. In terms of clinical staging, 48 (40%) participants were classified as stage 1, 36 (30%) as stage 2, and 36 (30%) as stage 3. Histopathological grading revealed mild dysplasia in 30 (25%), moderate dysplasia in 48 (40%), and severe dysplasia in 42 (35%) patients. Keratinization of the epithelium was observed in 84 (70%) participants, while epithelial thinning was noted in 60 (50%) participants. Dysplasia, a critical predictor of malignancy, was identified in 48 (40%) participants, while the remaining 72 (60%) showed no signs of dysplasia.

**Table 1:** Descriptive characteristics of the study population. Data presented in the form of n (%).

Parameters	Category	Frequency (n)	Relative frequency (%)
Sex	Male	90	75.00
	Female	30	25.00
Tobacco habit	Yes	102	85.00
	No	18	15.00
Smoking habit	Yes	66	55.00
	No	54	45.00
Alcohol habit	Yes	60	50.00
	No	60	50.00
Burning sensation	Yes	72	60.00
	No	48	40.00
Clinical stage	Stage 1	48	40.00
	Stage 2	36	30.00
	Stage 3	36	30.00
Histopathological grade	Mild	30	25.00
	Moderate	48	40.00
	Severe	42	35.00
Keratinization	Yes	84	70.00
	No	36	30.00
Epithelium thinning	Yes	60	50.00
	No	60	50.00
Dysplasia	Yes	48	40.00
	No	72	60.00

Age showed a significant difference, with younger participants being more likely to exhibit dysplasia (p = 0.001). Alcohol frequency was also significantly higher in the dysplasia group (p = 0.001). The VAS score for burning sensation was notably lower in the dysplasia group than in the non-dysplasia group, indicating a significant association (p = 0.003). Mouth opening was significantly reduced in the dysplasia group (p = 0.001), suggesting greater disease progression in the dysplasia group. Conversely, no significant differences were observed in tobacco use frequency (p = 0.941) or smoking frequency (p = 0.053) between the groups (Table 2).

**Table 2:** Descriptive statistics of the variables for dysplasia in oral submucous fibrosis (OSMF). VAS: Visual Analog Scale. *p-value < 0.05: significant, data is presented in the form of mean and standard deviation (SD).

Parameters	OSMF with dysplasia		OSMF without dysplasia		t value	p-value
	Mean	SD	Mean	SD		
Age (years)	30.37	3.95	34.91	4.54	3.99	0.001*
Tobacco frequency (pouch per day)	3.62	2.10	3.66	2.12	0.07	0.941
Smoking frequency (cigarettes per day)	3.62	2.44	2.16	3.01	1.97	0.053
Alcohol frequency (times per day)	1.50	0.88	0.41	0.77	5.02	0.001*
VAS score for burning sensation	3.62	2.97	5.91	2.60	3.15	0.003*
Mouth opening (mm)	19.25	3.22	22.1	1.89	4.42	0.001*

Logistic regression analysis identified significant predictors of dysplasia in the OSMF cases. Alcohol frequency (p = 0.0001), mouth opening (p = 0.0001), smoking frequency (p = 0.0001), and tobacco use frequency (p = 0.0007) were all significantly associated with dysplasia. Among these, alcohol consumption frequency exhibited the strongest association. Conversely, the VAS score for burning sensation showed no significant association with dysplasia (p = 0.5250) (Table 3).

**Table 3:** Logistic regression analysis with continuous variables for dysplasia. VAS: Visual Analog Scale. *p-value < 0.05: significant.

Variable	Logworth	Coefficient	t value	p-value
Alcohol frequency	15.447	0.319	9.52	0.001*
Mouth opening (mm)	8.155	-0.080	-6.26	0.001*
Smoking frequency	7.562	0.058	5.97	0.001*
Tobacco frequency	4.131	0.061	4.11	0.007*
VAS score	0.280	0.008	0.64	0.525

Logistic regression analysis of categorical variables identified significant associations of epithelial thinning (p = 0.000), alcohol consumption (p = 0.007), and smoking (p = 0.012) with dysplasia in OSMF cases. Epithelial thinning exhibited the strongest relationship, as indicated by the highest logworth and chi-square values. Conversely, no significant associations were observed for tobacco use (p = 0.998), burning sensation (p = 0.998), or keratinization (p = 0.998) (Table 4).

**Table 4:** Logistic regression analysis with categorical variables for dysplasia. VAS: Visual Analog Scale, LR: likelihood ratio. *p-value < 0.05: significant.

Variable	Logworth	Chi-square value (LR)	p-value
Epithelium thinning	8.772	36.303	0.001*
Alcohol habit	4.154	15.80	0.007*
Smoking habit	1.913	6.278	0.012*
Tobacco habit	0.001	0.000	0.998
Burning sensation	0.001	0.000	0.998
Keratinization	0.000	0.000	0.998

The predictor screening analysis ranked the factors contributing to dysplasia in the OSMF cases based on their relative importance. Alcohol emerged as the most influential predictor, contributing 29.70% to the overall model, with a proportion of 0.3481, securing the top rank. Epithelial thinning followed closely, with a contribution of 27.19% and a proportion of 0.3187, ranking second. Smoking ranked third, contributing 14.09% (portion 0.1651), while keratinization (6.67%, portion 0.0781) and burning sensation (6.21%, portion 0.0728) ranked fourth and fifth, respectively. Tobacco use was the least impactful predictor, contributing only 1.46% (portion 0.0171) to the model, and ranked sixth (Table 5).

**Table 5:** Predictor screening for dysplasia in oral submucous fibrosis (OSMF) cases.

Predictor	Contribution	Portion	Rank
Alcohol habit	29.6996	0.3481	1
Epithelium thinning	27.1925	0.3187	2
Smoking habit	14.0908	0.1651	3
Keratinization	6.6673	0.0781	4
Burning sensation	6.2123	0.0728	5
Tobacco habit	1.4629	0.0171	6

**Figure 2:**
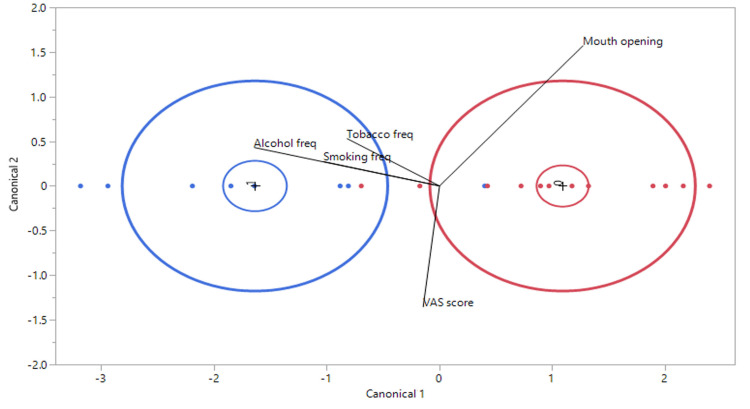
Discriminant analysis of independent factors distinguishing OSMF with dysplasia (blue) and OSMF without dysplasia (red). Freq: frequency. Image credit: This figure is an original creation of the authors.

The discriminant analysis with linear common variance plot shows the separation of two groups, OSMF without dysplasia (red) and OSMF with dysplasia (blue), based on canonical variables (Canonical 1 and Canonical 2). Canonical variables (Canonical 1 and Canonical 2) represent new dimensions derived from the original variables, such as alcohol frequency, smoking frequency, tobacco frequency, mouth opening, and VAS scores, through a process of linear combination. These dimensions were designed to maximize separation between the groups (red and blue). The variable mouth opening contributed most strongly in the positive direction along Canonical 1, aiding in distinguishing the group without dysplasia (red). Conversely, alcohol frequency, tobacco frequency, and smoking frequency were associated with the dysplasia (blue) group, and their contributions were evident in the negative direction along canonical 1. The VAS score showed minimal contribution. The wide separation of ellipses indicated effective discrimination between groups, with Canonical 1 being the primary dimension for differentiation. Canonical 2 offers minimal separation (Figure 2).

## Discussion

The results derived from this retrospective observational investigation yielded significant insights into the clinical and histopathological indicators of dysplasia in instances of OSMF. The correlation between alcohol consumption and dysplasia emerged as a pivotal finding in our study, underscoring the considerable impact of lifestyle choices on the advancement of OSMF. Alcohol consumption was the most potent predictor, accounting for nearly 30% of the total predictive model. This correlation is consistent with the hypothesis that alcohol functions as a co-carcinogen, intensifying the effects of additional risk factors such as tobacco and areca nut usage. Alcohol has the potential to augment the permeability of the oral mucosa, thereby facilitating the ingress of carcinogenic agents and contributing to the oxidative stress and inflammatory responses that are prevalent in OSMF. Furthermore, the incidence of alcohol consumption was markedly elevated in the dysplasia cohort, thereby reinforcing its significance as a pivotal factor in the dysplasia production and process of malignant transformation. These observations emphasize the need for targeted public health initiatives aimed at reducing alcohol intake among populations at elevated risk to alleviate the progression of OSMF. Our findings were in accordance with the study by Morse et al. [14] and contradict the findings of Lee et al. [15] and Nayak et al. [16]. Therefore, the role of alcohol consumption in OSMF progression remains controversial.

Epithelial atrophy, another critical predictor discerned in this investigation, exhibited a robust correlation with dysplasia. Histopathological analyses revealed that the attenuation of the epithelium functions as an essential biomarker of disease advancement, mirroring the fundamental pathological mechanisms, which encompass diminished cellular proliferation and heightened fibrosis. Incremental deterioration of epithelial thickness may jeopardize the integrity of the mucosal barrier, thereby amplifying vulnerability to carcinogenic agents and increasing the likelihood of malignant transformation. This finding emphasizes the necessity of routine histopathological evaluations in cases of OSMF to recognize epithelial atrophy as an early sign of dysplasia and prospective malignancy. Similar results were reported in previous studies [17,18]. Sarode et al. [19] posited that increased shedding of superficial cellular structures exists due to the diminished protective effect of a salivary mucous gel. Consequently, despite the heightened proliferative activity, accelerated exfoliation results in atrophic epithelium.

Based on the discriminant analysis, smoking has been identified as a prominent predictor, occupying the third position in its influence on dysplasia within the context of OSMF. The involvement of smoking in the process of oral carcinogenesis has been extensively substantiated by empirical research, which underscores its role in promoting oxidative stress, inducing DNA damage, and instigating modifications in cellular signaling pathways [16]. The cumulative effect of smoking, particularly when it is concomitantly present with additional risk factors such as the consumption of alcohol and areca nuts, engenders a synergistic effect that expedites the advancement of OSMF [15,16]. Tobacco undergoes metabolic activation through the action of cytochrome P450 enzymes, resulting in the formation of N-nitronicotinoids, which possess the capacity to induce DNA damage and subsequently contribute to the development of potentially malignant pathologies, culminating in oral carcinoma [20].

One noteworthy observation was the absence of a statistically significant correlation between the burning sensation, a prevalent symptom in OSMF, and the presence of dysplasia. Although burning sensation is frequently perceived as a marker of disease severity, its nonexistence within the dysplasia cohort implies that it may not serve as a dependable predictor of malignant transformation [21]. This observation calls into question traditional diagnostic methodologies that emphasize symptomatic assessment and highlights the necessity for objective histopathological and molecular markers to evaluate the malignancy risk associated with OSMF. Similarly, keratinization, which was noted in a considerable proportion of subjects, exhibited a minimal impact on the prediction of dysplasia. This indicates that keratinization may signify the epithelial adaptive response to persistent irritation; it does not constitute a direct indicator of dysplasia or malignant potential [22].

The investigation further elucidated the notable correlation between diminished oral aperture and dysplastic conditions, with instances of dysplasia exhibiting significantly restricted mouth opening. This clinical parameter signifies advancing fibrosis and rigidity of the oral tissues, which are essential characteristics of advanced OSMF. The reduction in oral aperture not only detrimentally affects the quality of life but also functions as a pivotal clinical indicator of disease advancement and the associated risk of dysplasia. Consequently, timely recognition and intervention of restricted mouth opening are imperative for averting additional disease progression and enhancing patient prognoses [1,23].

From a methodological perspective, the use of discriminant analysis to separate OSMF cases with and without dysplasia provides valuable insights into the relative contributions of various predictors. The canonical variables derived from this analysis effectively distinguished between the two groups, with increased alcohol, smoking, and tobacco frequency contributing to the dysplasia group. Similarly, reduced mouth opening has also been associated with increased dysplastic changes in the OSMF [1,5,16,23].

Clinical implications and future recommendations

The results of this investigation have considerable ramifications for the clinical management and prophylaxis of OSMF. The discernment of smoking and tobacco chewing behaviors, along with epithelial atrophy, as the most significant predictors of dysplasia, underscores the necessity for focused screening and intervention initiatives within populations identified as high risk. Public health initiatives that emphasize the cessation of these detrimental behaviors could substantially reduce the likelihood of dysplasia and malignant progression in patients with OSMF. Moreover, systematic histopathological examinations, which encompass the evaluation of epithelial thickness and keratinization, should be incorporated into conventional diagnostic frameworks to facilitate early identification and risk assessment.

In addition to the clinical and public health ramifications, this study emphasizes the need for further investigation into the molecular mechanisms that contribute to dysplasia in OSMF. The significance of molecular biomarkers, including p53 mutations, anomalies in cell cycle regulation, and the expression of inflammatory cytokines, necessitates further investigation to improve diagnostic precision and facilitate the development of targeted therapeutic strategies. Progress in molecular diagnostics has the potential to aid the early identification of high-risk individuals, thus allowing for timely interventions and a reduction in the incidence of oral cancer.

Limitations of the study

This study was subject to multiple constraints. Given its retrospective nature, the study depended on previously documented information, which may have resulted in incomplete or erroneous records. The calculated sample size, while methodologically sound, may not adequately reflect the varied demographics and behaviors of populations affected by the OSMF. The absence of standardized histopathological parameters for dysplasia classification can result in interpretative discrepancies. Additionally, this study did not incorporate genetic or environmental variables that may affect the trajectory of the disease. The lack of analysis of molecular markers restricts our ability to thoroughly examine the fundamental mechanisms involved. Future prospective research involving larger and more diverse cohorts alongside molecular assessments is essential.

## Conclusions

This investigation elucidates the role of alcohol intake, epithelial thinning, and tobacco use as critical determinants of dysplastic changes in OSMF. Limited mouth opening also exhibited a robust correlation with disease progression. Although keratinization and burning sensation were prevalent, they displayed a negligible relationship with the risk of dysplasia. These results underscore the necessity for regular histopathological assessments and focused lifestyle modifications, particularly concerning alcohol consumption and smoking behaviors, to reduce the likelihood of malignant transformation. Subsequent studies that integrate molecular biomarkers and prospective methodologies are imperative to improve diagnostic precision and formulate tailored management approaches for individuals with high-risk OSMF.
